# Information theory of metasurfaces

**DOI:** 10.1093/nsr/nwz195

**Published:** 2019-11-27

**Authors:** Haotian Wu, Guo Dong Bai, Shuo Liu, Lianlin Li, Xiang Wan, Qiang Cheng, Tie Jun Cui

**Affiliations:** 1 State Key Laboratory of Millimeter Waves, Southeast University, Nanjing 210096, China; 2 School of Physics and Astronomy, University of Birmingham, Birmingham B15 2TT, UK; 3 State Key Laboratory of Advanced Optical Communication Systems and Networks, Department of Electronics, Peking University, Beijing 100871, China

**Keywords:** metasurfaces, differential entropy, radiation information bounds, maximum orthogonal radiation patterns, information invariance

## Abstract

We propose a theory to characterize the information and information processing abilities of metasurfaces, and demonstrate the relation between the information of the metasurface and its radiation pattern in the far-field region. By incorporating a general aperture model with uncertainty relation in *L*^2^-space, we propose a theory to predict the upper bound of information contained in the radiation pattern of a metasurface, and reveal the theoretical upper limit of orthogonal radiation states. The proposed theory also provides guidance for inverse design of the metasurface with respect to given functionalities. Through investigation of the information of disordered-phase modulated metasurfaces, we find the *information invariance* (1−γ, where *γ* is Euler's constant) of chaotic radiation patterns. That is to say, the information of the disordered-phase modulated radiation patterns is always equal to 1−*γ*, regardless of variations in size, the number of elements and the phase pattern of metasurface. This value might be the lower bound of radiation-pattern information of the metasurface, which can provide a theoretical limit for information modulation applications, including computational imaging, stealth technologies and wireless communications.

## INTRODUCTION

Metamaterial has a pivotal role in regulating photons, allowing manipulation of lights to realize intriguing functionalities, such as negative refraction, perfect imaging and invisibility cloaking [1–4]. The latest development of metasurface, with reduced dimensionality, has exceptional abilities for controlling the flow of lights [5–7]. Subwavelength-scale particles of metasurfaces can couple incident waves to free space with controllable amplitudes and phases, such that the transmitted waves can be flexibly manipulated by designing the response and distribution of meta-particles. Recently, investigations of metasurfaces have been extended from material science to the digital and information category [8–10], in which the metasurfaces can be considered as information processors in physical hardware layers and have useful applications such as in computational imaging [11,12], wireless communications [13,14] and performance of mathematical operations [15,16]. However, from an information perspective, the study of metasurfaces needs a general theory for guidance.

In this work, we propose a general information theory of metasurfaces to characterize the information processing ability. Based on the theory, we aim to predict the upper bound of information contained in the radiation pattern of metasurfaces, and to reveal the theoretical upper limits of orthogonal radiation states. In particular, we investigate the information of disordered-phase modulated metasurfaces and find the information invariant property of their chaotic radiation patterns, which may give the lower bound of the radiation-pattern information. The proposed theory will provide theoretical limits for information modulation applications of metasurfaces, including computational imaging, stealth technology and wireless communications.

## RESULTS AND DISCUSSION

To analyze the metasurface from an information perspective, we introduce a *general aperture model*, which is used to characterize the information and information processing ability of metasurface. The general aperture model suggests that each metasurface particle is considered to be a small aperture with uniform amplitude and phase, such that the metasurface is a general aperture consisting of many small apertures. To illustrate this idea, the function ${\varphi _{\mathit {ij}}}({\boldsymbol{r}}\!)$ is adopted to represent a rectangular-shaped metasurface particle with the area *s* = *a* × *b*, which can be expressed as:

(1) \begin{eqnarray*}{\varphi _{\mathit {ij}}}({\boldsymbol{r}}\!) &=& {A_{\mathit {ij}}}{e^{\!j{\theta _{\mathit {ij}}}}} \cdot \Pi \left[\frac{{x - a(i - 1)}}{a}\right]\cdot \nonumber\\ && \Pi \left[\frac{{y - b(j - 1)}}{b}\right],\end{eqnarray*}

where Π(*t*) is the rectangular function, and ${A_{\mathit {ij}}}$ and ${\theta _{\mathit {ij}}}$ are the amplitude and phase responses of the *ij*^th^ particle, respectively. The aperture function of the metasurface can be expressed by a combination of these small apertures:

(2) \begin{equation*}{\varphi _A}{\rm{(}}{\boldsymbol{r}}\!{\rm{) = }}{{\sum\limits_{i = 1}^{{N_x}} {\sum\limits_{j = 1}^{{N_y}} {{\varphi _{\mathit {ij}}}({\boldsymbol{r}})} } } / {{{\left(a \cdot b \cdot \sum\limits_{i = 1}^{{N_x}} {\sum\limits_{j = 1}^{{N_y}} {A_{\mathit {ij}}^2} }\right )}^{\frac{1}{2}}}}},\end{equation*}

where *N_x_* and *N_y_* are the numbers of particles along the *x* and *y* directions, and the denominator term is introduced to normalize the square integral of the aperture function. The aperture function is formulated by the electromagnetic response of the metasurface, which contains both amplitude and phase distribution information for the metasurface. It should be noted that coupling effects generally exist among neighborhood meta-particles, such that the overall effective electric field distribution of the metasurface is a combination of the single-particle generated field and coupled field. As a result, ${A_{\mathit {ij}}}$ and ${\theta _{\mathit {ij}}}$ in Eqs 1 and 2 should be interpreted as the effective amplitude and phase responses of the meta-particles, which have taken into account the coupling effects [17].

The electric-field distribution on the metasurface is a quasi-determined quantity, with negligible uncertainty resulting from the zero-point energy fluctuations [18]. To characterize the information properties of the metasurface and its corresponding radiation pattern, we first invoke the close analogy between the oscillating electric field and Schrödinger's wave function. It has been established that both cases can be converted into generalized eigenvalue problems with Hermitian operators expressed as: ${{\boldsymbol{H}}^{\!\!1}} = \nabla \times \nabla \times $ and ${{\boldsymbol{H}}{\!^2}} = - \frac{{{\hbar^{\!2}}}}{{2m}}{\nabla ^2} + V\!({\boldsymbol{r}} )$, respectively. The two solutions (eigen-functions) can be decomposed into harmonic modes that oscillate with the phase factor ${e^{\!jwt}}\ $[19].

Based on the similar algebraic structure of these two problems, we propose consideration of the aperture function of metasurface, ${\varphi _A}( {\boldsymbol{r}}\!)$, which is proportional to the electric field distribution of metasurface, as an effective Schrödinger's wave function, such that the square of the normalized wave function $\varphi _A^2( {\boldsymbol{r}}\! ) = \varphi _A^*( {\boldsymbol{r}}\! ) \times {\varphi _A}( {\boldsymbol{r}}\! ) = {| {{\varphi _A}( {\boldsymbol{r}}\! )} |^2}$ can be considered as a density function describing the energy/photon distribution on the metasurface plane. We note that the density function of the metasurface $\varphi _A^2( {\boldsymbol{r}}\!)$ is pre-normalized, hence it can be further interpreted as an effective probability density function (PDF). The close analogy between the oscillating electric field and quantum wave function allow us to study the metasurface from the perspective of information science.

### Boltzmann-Shannon entropy

We adopt Boltzmann-Shannon entropy to characterize distribution of energy on the metasurface [20]. As discussed above, when a monochromatic plane wave impinges on a metasurface, PDF of energy distribution in position space can be expressed as ${P_1}( {\boldsymbol{r}}\! ) = \varphi _A^2( {\boldsymbol{r}}\! )$. Then, *differential entropy* can be adopted to characterize the position uncertainties of energy on the metasurface plane, expressed as:

(3) \begin{eqnarray*}H({\boldsymbol{r}}\!)&=& H({P_1}({\boldsymbol{r}}\!)) = H(\varphi _A^2({\boldsymbol{r}}\!))\nonumber\\ &=& - \int{{\int{{\varphi _A^{\rm{2}}({\boldsymbol{r}}\!)\!\ln }}}}\varphi _A^{\rm{2}}({\boldsymbol{r}}\!)d{r^{\!2}}.\end{eqnarray*}

Once the size of metasurface is determined, the differential entropy of the metasurface aperture is restricted by an upper bound value of ln*S*, where *S* is the total size of the metasurface. Therefore, we define the information of the metasurface as reduction of uncertainty from the maximum, which can be derived from Eqs 2 and 3 as:

(4) \begin{eqnarray*}{I_1} &=& I\!({\boldsymbol{r}}\!) = - \Delta {H_1}({\boldsymbol{r}}\!) = H{({\boldsymbol{r}}\!)_{\max }} - {H_1}({\boldsymbol{r}}\!) \nonumber\\ &=& \ln\! {N_x}{N_y}{\rm{ + }}\sum\limits_{i = 1}^{{N_x}} \sum\limits_{j = 1}^{{N_y}} {c_{\mathit {ij}}^{\!2}} \ln c_{\mathit {ij}}^{\!2},\end{eqnarray*}

where$\ {c_{\mathit {ij}}} = {( {A_{\mathit {ij}}^2/\mathop \sum \nolimits_{i = 1}^{{N_x}} \mathop \sum \nolimits_{j = 1}^{{N_y}} A_{\mathit {ij}}^2} )^{\frac{1}{2}}}$. The detailed derivations are given in the supplementary data. One of the advantages of the proposed definition, compared with the differential entropy, is that the information term is strictly non-negative and scale-invariant, such that different choices of unit would not affect the final results of information.

Heisenberg's uncertainty principle expresses the indeterminacy of two observables with the computation rule [**α**, **β**]≠0 in terms of the second moments [21,22]. Later studies of *L*^2^-space [23,24] provide another form of the uncertainty with respect to the non-commuting observables (**α** and **β**) in terms of the differential entropy as:

(5) \begin{equation*}\Delta {{\boldsymbol \alpha }} + \Delta {{\boldsymbol \beta }} \ge n{\rm{(1 + ln}}\pi),\end{equation*}

where ${{\Delta }}{{\bf T }} = - {\small {int}} P\!({{\boldsymbol \tau }}){\rm ln}\!P\!({{\boldsymbol \tau}})d{\tau}^n$, and the term *P*(***τ***) is the PDF of random variable **T**, and Δ**T** is the differential entropy of **T** in *n*-dimensional space.

It has been established that the electric far-field $E\!( {\boldsymbol{k}} )$ in the wave-vector space (*k*-space) is the Fourier transform of electric field distribution on the metasurface ${\varphi _A}( {\boldsymbol{r}} ){\rm{\ }}$[9], thus ***r*** and ***k*** could be considered as two observables with the commutation rule of $[ {{{{\boldsymbol{\hat{r}}}}_m}\!,{{{\boldsymbol{\hat{k}}}}_n}} ] = i\!{\delta _{mn}}$ (*m*, *n* = 1, 2), where ${\boldsymbol{\hat{r}}}$ and ${\boldsymbol{\hat{k}}}$ are the corresponding operators [25]. Therefore, the differential entropy of the far-field energy density function in the *k*-space can be expressed as:

(6) \begin{eqnarray*}H({\boldsymbol{k}}) &=& H({P_2}({\boldsymbol{k}})) = H(f\!({\boldsymbol{k}}))\nonumber\\ &=& - \int{{\int{{f\!({\boldsymbol{k}})\!\ln\!\! f\!({\boldsymbol{k}})}}d{k^2}}},\end{eqnarray*}

where $f\!( {\boldsymbol{k}} ) = {\rm{\alpha }}{E^{\!2}}( {\boldsymbol{k}} )$ is the normalized far-field energy density distribution function, and α is a constant coefficient to normalize the function. Based on inequality 5, the term *H*(***k***) would be constrained by the uncertainty relation as:

(7) \begin{eqnarray*}H({\boldsymbol{k}})&=& H(\!f\!({\boldsymbol{k}})) \ge \ln\! {(\pi {\rm{e}})^2} - H({{\bf r}})\nonumber\\ &=& {\rm{ln}}\frac{{{\pi ^2}{e^2}}}{{ab}}{\rm{ + }}\sum\limits_{i = 1}^{{N_x}} {\sum\limits_{j = 1}^{{N_y}} {c_{\mathit {ij}}^2} } \ln\! c_{\mathit {ij}}^2.\end{eqnarray*}

Similarly, the information of radiation pattern (*I*_2_) in the *k*-space is defined by reduction of the wave-vector uncertainty from the maximum value as ${I_2} = H{( {\boldsymbol{k}} )_{max}} - H( {\boldsymbol{k}} )$, satisfying the relation that:

(8) \begin{eqnarray*}{I_2} &=& I\!({\boldsymbol{k}}) = H{({\boldsymbol{k}})_{\max }} - H({\boldsymbol{k}}) \le \ln \frac{{ab{k^2}}}{{\pi {e^2}}}\nonumber\\ && - \sum\limits_{i = 1}^{{N_x}} {\sum\limits_{j = 1}^{{N_y}} {c_{\mathit {ij}}^2} } \ln\! c_{\mathit {ij}}^2.\end{eqnarray*}

Thus the information relation between the metasurface and its radiation pattern can be derived from Eq. 4 and inequality 8 as:

(9) \begin{equation*}{I_1}{\rm{ + }}{I_2}{\rm{ = }}I\!({{\bf r}}) + I\!({\boldsymbol{k}}) \le \ln \left(\frac{{4\pi \cdot S}}{{{e^2}{\lambda ^2}}}\right),\end{equation*}

where $S = {N_x} \times {N_y} \times a \times b$ is the size of the metasurface, and *λ* is the wavelength of the electromagnetic waves. This inequality implies an important fact, that the total information of a metasurface and its radiation pattern has an upper bound. Detailed derivations of inequalities 7 to 9 are given in the supplementary data. The information of radiation pattern is defined based on the concept of normalized wave function and differential entropy, which is quite different from the previously established concept of image entropy [9], as discussed in the supplementary data. It is noteworthy that inequality 9 is valid as long as the Fourier transform relation holds between the aperture function ${\varphi _A}( {\boldsymbol{r}}\!)$ and electric far-field $E\!({\boldsymbol{k}} )$, otherwise the proposed theory cannot be applied (e.g. when the incident wave is converted to a surface wave by the metasurface [26]). In addition, inequality 9 is formulated for a specified radiation state with fixed state parameters (e.g. fixed frequency and polarization). Therefore, if multiple states (e.g. two orthogonal polarized states) are considered, inequality 9 should be modified by labeling the state parameter(s) for clarification.

**Figure 1.**
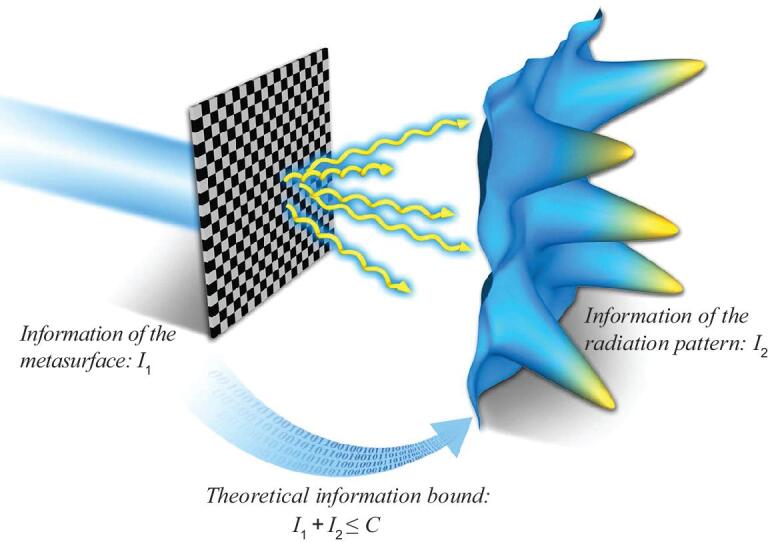
Schematic of information relation between the metasurface and its radiation pattern.

**Figure 2.**
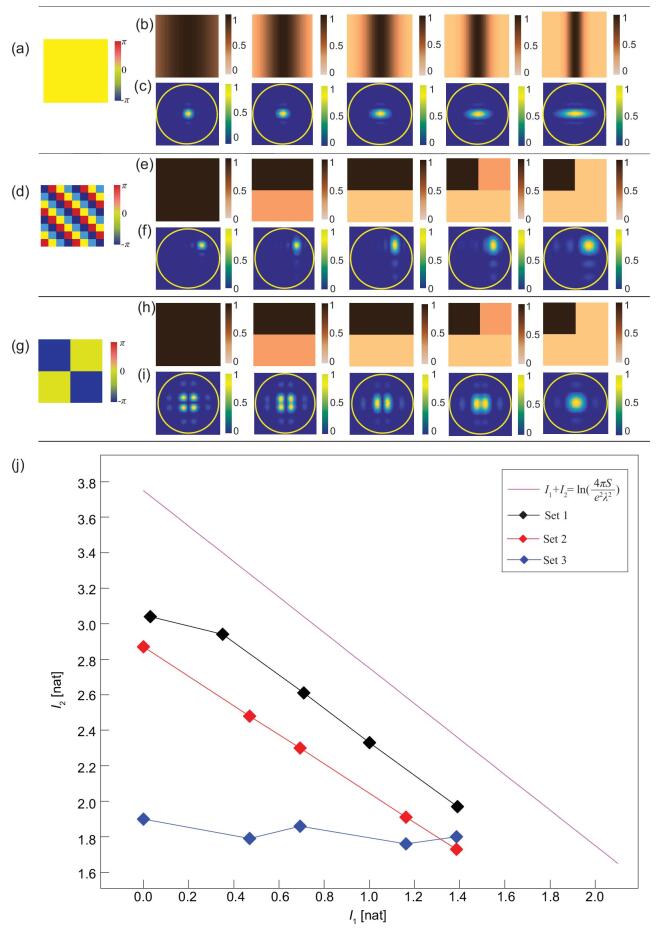
Phase (a, d, g) and amplitude (b, e, h) distributions of the three sets of metasurface samples. (c, f, i) Normalized radiation patterns generated by the three sets of metasurface samples. (j) Calculated results of the information relation between the metasurface samples and their radiation patterns, and the upper bound.

Based on the above analyses, a bridge has been built to connect the information of the metasurface and its radiation pattern, as sketched in Fig. 1. To illustrate these results, we first analyze three simple sets of metasurfaces containing 40 × 40 subwavelength particles, in which each particle occupies an area of *λ*/8 × *λ*/8. The phase distributions of the metasurface samples are plotted in Fig. 2a, d and g, respectively, and the amplitude distributions are plotted in Fig. 2b, e and h, respectively, in which five different cases are considered for each phase.

In the first set of metasurface samples, the phase distribution is uniform across the aperture (Fig. 2a), and the amplitude distributions are 1-dimensional Gaussian functions with different standard derivations (Fig. 2b). The calculated radiation patterns with respect to different amplitude distributions are plotted in the *k*-space (*k_x_*, *k_y_*), as shown in Fig. 2c. We notice that the radiation patterns become more spread in the horizontal direction as the contrast ratio of the aperture function increases in the same direction. The phase distribution of the second set of metasurfaces is set as a gradient function (Fig. 2d), and the radiation patterns generated by different staircase-amplitude functions (Fig. 2e) are plotted in Fig. 2f. As the contrast ratio of the aperture function increases in both directions, the radiation pattern also becomes more spread over the *k*-space. In the third set of samples, the phase distribution of the metasurface is ‘0 −π/−π 0’ (Fig. 2g), and the radiation patterns generated by different amplitude distributions (Fig. 2h) are plotted in Fig. 2i. The results show that the radiation pattern blurs in the vertical direction as the contrast ratio of the two rectangular regions increases, resulting in the original four discernible bright spots to be gradually fused to two elliptical shapes. When the contrast ratio of the aperture function continues to increase horizontally, the two horizontal bright spots finally merged into one indiscernible spot (Fig. 2i). More results with different amplitude and phase patterns can be found in the supplementary data. The relation between the information of metasurfaces and radiation patterns is presented in Fig. 2j. We note clearly that the calculated results of *I*_1_ and *I*_2_ satisfy the requirement determined by inequality 9, showing that the total information of the three cases is below the theoretical upper limit.

From the blue curve (Set 3), we also note that the information of the radiation pattern does not decrease monotonically with increasing information on the metasurface, because the phase distribution plays a key role in generating radiation patterns with different information (*I*_2_). According to inequality 9, the upper bound of *I*_2_ decreases linearly as *I*_1_ increases, which would cause information on the radiation pattern (*I*_2_) to tend to decline as information on the metasurface (*I*_1_) increases in general.

### Maximum number of orthogonal radiation states and information modulation in *k*-space

**Figure 3.**
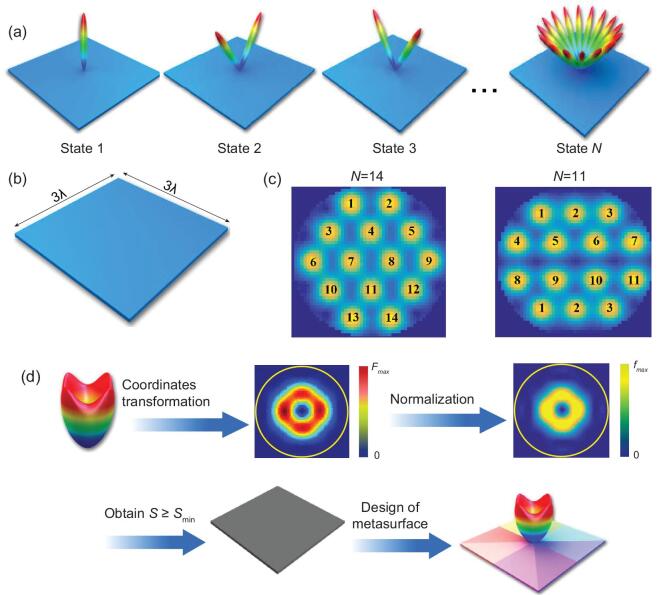
(a) A set of orthogonal radiation states in the *k*-space. (b) The size of metasurface. (c) Two examples of quasi-orthogonal radiation patterns in the *k*-space. (d) The inverse design procedure of metasurface with respect to certain radiation patterns.

A large number of radiation patterns can be generated from a single digitally programmable metasurface by altering the states of the implemented active devices with controlled circuits [8]. In these realizable radiation patterns, orthogonal states (Fig. 3a) are preferred for information modulation and processing, for example in computational imaging [11] and communications. Here, leveraging the proposed information theory of the metasurface, an insightful approach is presented to characterize the theoretical maximum of the orthogonal radiation states without complicated calculations. First, suppose a set of normalized orthogonal far-field radiation patterns (${f^{\!i}}\!( {\boldsymbol{k}} )$) with number *N* already obtained, and the set of radiation patterns satisfies the vector product relation as:

(10) \begin{equation*}(\!{f^{\!{i}}}({\boldsymbol{k}}),{f^{\!j}}\!({\boldsymbol{k}})) = \int{{{f^{\!i}}\!({\boldsymbol{k}}) \cdot {f^{\!j}}\!({\boldsymbol{k}})}}d\!{\boldsymbol{k}}{\rm{ = }}{\delta _{\mathit {ij}}},\end{equation*}

where ${\delta _{\mathit {ij}}}$ is the Kronecker function. Suppose that the radiation pattern with information *I*_2_ occupies an area of *C* in the *k*-space, such that *I*_2_ can be expressed as:

(11) \begin{eqnarray*}{I_2} &=& I\!({k_x}\!,{k_y}\!) = \ln\! \pi {k^2}\nonumber\\ &&+ \int \!\!\!\int_C {P\!({k_x}\!,{k_y}\!)\!\ln [P\!({k_x}\!,{k_y}\!))]d{k_x}d{k_y}}.\nonumber\\ \end{eqnarray*}

Then we consider another radiation pattern with information $I_2^C$ uniformly distributed in the same region of *C*, which would carry the minimum information as:

(12) \begin{equation*} I_2^C = \ln ({{\pi {k^2}} / C}) \le {I_2}. \end{equation*}

Therefore, the occupation area of each radiation pattern in the *k*-space must satisfy the condition that: ${C^i} \ge \pi {k^2}{e^{ - I_2^i}}$. Additionally, summation of ${C^i}\ $should not outrange the total occupation area in the *k*-space, which can be expressed as:

(13) \begin{equation*}\sum\limits_{i = 1}^N {\pi \cdot {k^2} \cdot {e^{ - I_2^i}}} \le \sum\limits_{i = 1}^N {{C^i}} \le \pi \cdot {k^2}.\end{equation*}

On the other hand, the information $I_2^i$ on each radiation pattern is restricted by inequality 9 as: $I_2^i \le I_1^i + I_2^i \le {\rm{ln}}( {\frac{{4\pi S}}{{{e^2}{\lambda ^2}}}} )$. Therefore, the maximum number (*N*) of the orthogonal radiation patterns can be deduced as:

(14) \begin{equation*}N \le \left[ {\frac{{4\pi \cdot S}}{{{e^2}{\lambda ^2}}}} \right],\end{equation*}

where [] denotes the rounding down symbol.

**Figure 4.**
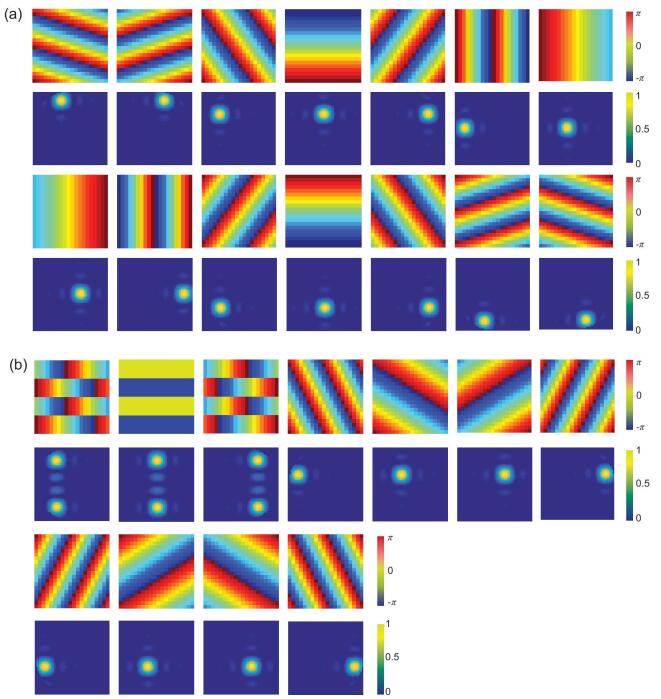
(a, b) Phase distribution and the corresponding radiation patterns of two sets of metasurface samples.

For a simple demonstration, we consider a metasurface with size set as ${\rm{S}} = 3{\rm{\lambda }} \times 3{\rm{\lambda }}$, shown in Fig. 3b, in which the maximum number of the orthogonal patterns is restricted by inequality 14 as: $N \le [ {\frac{{4\pi \times 3\lambda \times 3\lambda }}{{{e^2}{\lambda ^2}}}} ] = 15$. Two sets of quasi-orthogonal radiation patterns are presented in Fig. 3c. We observe that the different radiation patterns share negligible overlaps in the *k*-space, such that each radiation pattern can be unambiguously distinguished. The phase distributions of the metasurfaces corresponding to these radiation patterns are presented in Fig. 4. We notice that the numbers of orthogonal radiation patterns realized in both examples meet the requirement set by inequality 14, which are consistent with theoretical predictions.

### Inverse design of metasurfaces

In designing the metasurface, it is vital to choose a suitable size with respect to different functions. The proposed theory can provide guidance to determine the size of metasurface for required radiation pattern(s). To begin, a mapping process should be carried out to transform the required radiation pattern(s) into the coordinates [*k_x_*, *k_y_*], as sketched in Fig. 3d. Then, the obtained radiation pattern *F*(*k_x_*, *k_y_*) should be further normalized to become a probabilistic function, *f*(*k*, *k_y_*). In some cases, multiple radiation patterns $({f^{\!1}},{f^{\!2}},{f^{\!3}} \ldots )$ are required to design programmable metasurfaces. Therefore, the lower bound of metasurface size can be derived from Eq. 11 and inequality 14 as:

(15) \begin{eqnarray*}S &\ge& \frac{{{e^2}{\lambda ^2}}}{{4\pi }}\max [\exp (\ln\! \pi {k^2}\nonumber\\ &&{\rm{ + }}\int{{\int{{{f^{\!i}}\!({k_x}\!,{k_y}\!)}}}}\!\ln\! \!{f^{\!i}}\!({k_x}\!,{k_y}\!)d{k_x}d{k_y}\!)],\nonumber\\ \end{eqnarray*}

where ${f^{\!i}}$ is the ${i^{\!th}}$ radiation pattern. Detailed derivations are given in the supplementary data. The above analysis implies that the size of metasurface must be larger than the value predicted by inequality 15, otherwise it would be impossible to realize the required radiation pattern(s) no matter what design strategies were adopted.

### Information of disordered-phase modulated metasurfaces

**Figure 5.**
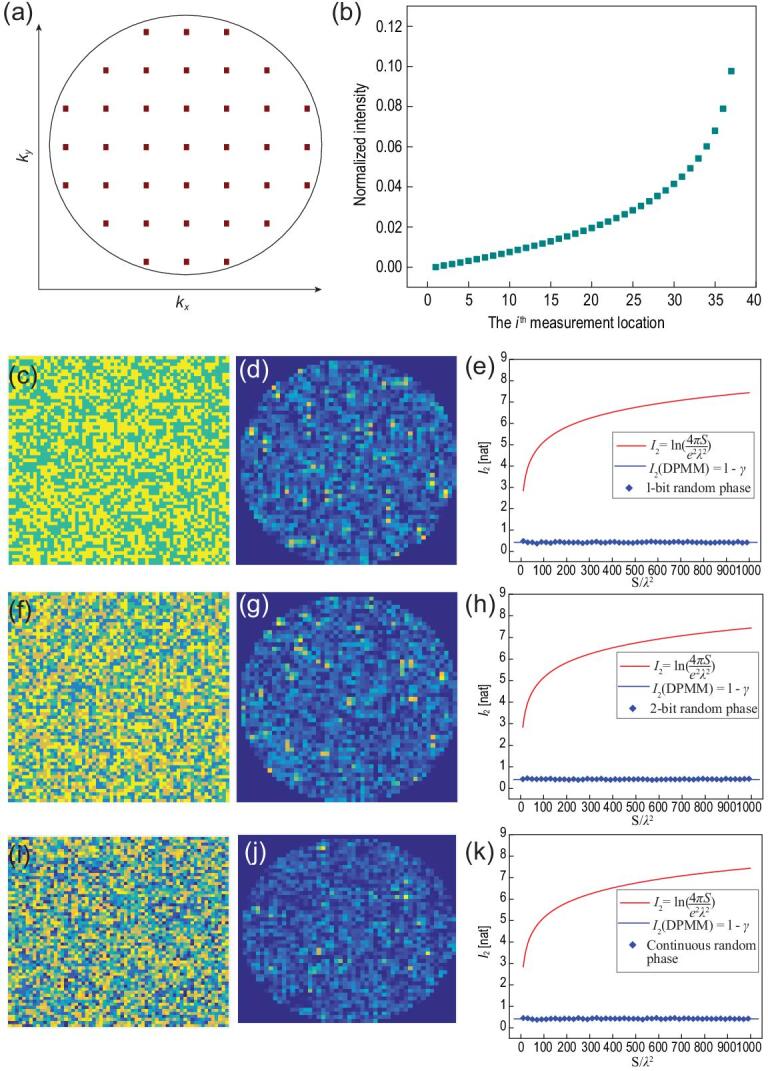
(a) A set of measurement locations in the $[ {{k_x},{k_y}} ]$ space with *M* = 37. (b) Theoretical intensity distributions of the measurement points. (c, d) A sample of 1-bit disordered-phase pattern of the metasurface and the generated far-field intensity distribution. (e) Theoretical and calculated results of *I*_2_ with respect to different sizes and phase patterns of metasurfaces. (f, g) A sample of 2-bit disordered-phase pattern of metasurface and the generated far-field intensity distribution. (h) Theoretical and calculated results of *I*_2_ with respect to different sizes and phase patterns of metasurfaces. (i, j) A sample of continuous disordered-phase pattern of the metasurface and the generated far-field intensity distribution. (k) Theoretical and calculated results of *I*_2_ with respect to different sizes and phase patterns of metasurfaces.

The metasurfaces that are modulated with disordered-phase distributions can diffuse the incoming electromagnetic wave into speckle-shaped far-field patterns, which have been used widely in stealth technologies [8,27] and computational imaging systems [11,12]. The information on the radiation patterns (although chaotic) can be distinctively described, leveraging the proposed theory. To begin, we consider an isotropic metasurface with the reflection coefficient expressed as:

(16) \begin{equation*}t = \alpha \cdot {e^{\!i\! \cdot \theta (x,y)}},\end{equation*}

where the amplitude response *α* is a constant, while the phase response *θ*(*x*, *y*) is randomly distributed on the metasurface. With superposition of the disorder-distributed complex near fields, the electric far-field $E\!({\boldsymbol{k}} )\ $is approximated as a complex Gaussian random variable with mean value of zero from the central limit theorem [28,29], featuring a radiation PDF for the far-field intensity that obeys the exponential statistic as:

(17) \begin{equation*}P\!({{g}}\!) = \frac{1}{\lambda }{e^{ - \frac{1}{\lambda } \cdot g}},\end{equation*}

where *g* represents the far-field intensity, and *λ* is an undetermined parameter. Next, we consider the case with *M* points uniformly distributed in the *k*-space (Fig. 5a), which gives the intensity distribution uncertainty of ln*M*. The obtained intensity data from the measurements in the far-field region should satisfy the PDF of Eq. 17, with the parameter *λ* substituted with 1/*M* as:

(18) \begin{equation*}P\!(M,g\!) = M{e^{ - M \cdot g}}.\end{equation*}

To find the far-field intensity distribution at these *M* locations, we divide the obtained PDF into *M* equally sized portions, in which each integral of the probability function *P*(*M*, *g*)d*g* equals 1/*M*. Such a partition process generates a set of points with number *M* (*i* = 1, 2, 3…*M*), which approximates the far-field energy distribution at the measurement locations, obeying the relation that:

(19) \begin{equation*}\int_{0}^{{\frac{i}{M}}}{{M \cdot {e^{ - M \cdot {g_{\!M}}\!(i)}}}} = \frac{{i - 1}}{M}\,\left( {i = 1,{\rm{ }}2 \ldots ,M} \right),\end{equation*}

where ${g_{\!M}}( i )$ is the intensity of the radiation pattern at the ${i^{th}}\ $measurement location. Thus, the far-field intensity function can be solved by Eq. 19:

(20) \begin{equation*}{g_{\!M}}(i) = - \frac{1}{M}\ln \frac{{M - i + 1}}{M}\,\left( {i = 1,{\rm{ }}2 \ldots ,M} \right),\end{equation*}

as plotted in Fig. 5b. When the number of measurement points *M* approaches infinity, Eq. 20 can provide an accurate prediction of the radiated energy distribution of the disordered-phase modulated metasurface (DPMM), and its far-field information can be derived from Eqs 11 and 20 as:

(21) \begin{eqnarray*} &&{{I_2}(\rm {DPMM}) = H{({\boldsymbol{k}})_{\max }} - H({\boldsymbol{k}})}\nonumber\\ &&=\mathop {\lim }\limits_{M \to \infty } \ln\! M - \mathop {\lim }\limits_{M \to \infty } \sum\limits_{i = 1}^M { - {g_{\!M}}(i)} \cdot \ln [{g_{\!M}}(i)]\nonumber\\ &&= - \int_{0}^{1}{{\ln (1 - \tau )\ln ( - \ln (1\! -\! \tau ))d\tau }} = 1 - \gamma,\nonumber\\ \end{eqnarray*}

where *γ* is Euler's constant (γ≈0.5772). A detailed derivation of Eq. 21 is given in the supplementary data. The above results, surprisingly, lead to the conclusion that the information on the chaotic far-field pattern of DPMM is a constant, regardless of the metasurface size, the number of metasurface elements and the phase-distribution pattern.

**Figure 6.**
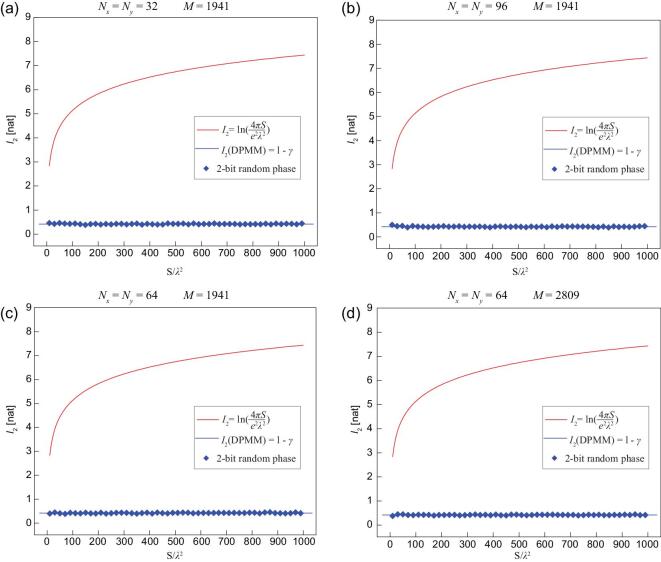
(a-d) Theoretical and calculated results of the disordered-phase modulated far-field information *I*_2_ with respect to different numbers of metasurface elements (*N_x_* and *N_y_*) and measurement locations (*M*).

To verify the above analysis, we calculate the information of far-field patterns generated by three sets of DPMM samples: 1-bit digital coding phases [8] with each *θ*(*x*, *y*) taking on one of the values of 0 and *π* randomly, as shown in Fig. 5c; 2-bit digital coding phases with each *θ*(*x*, *y*) taking on one of the values of 0, *π*/2, *π*, and 3/2*π* randomly, as shown in Fig. 5f; and continuous random phase variable *θ*(*x*, *y*) described by a uniform probability distribution of *P_θ_* = 1/2*π*, 0≤θ<2*π*, as shown in Fig. 5i. In these figures, the total number of metasurface elements is set as *N_x_* × *N_y_* = 64 × 64, and the number of measurement locations is *M* = 1941. Each set of these experiments involves 50 different phase patterns, associated with the size of metasurface ranging from *S* = 10*λ*^2^ to *S* = 1000*λ*^2^. The calculated information results of the disordered-phase radiation patterns are illustrated in Fig. 5e, h, and k, respectively, and the far-field information results with respect to different parameters (*N*_x_, *N*_y_ and *M*) are given in Fig. 6. It is evident that information on the speckle-like radiation patterns matches the theoretical prediction of Eq. 21 perfectly, convincingly demonstrating the information invariance of the chaotic far-field patterns.

The information on radiation pattern (${I_2} = H{( {\boldsymbol{k}} )_{max}} - H( {\boldsymbol{k}} )$) measures the entropic difference between the generated radiation pattern $H( {\boldsymbol{k}} )$ and the omnidirectional radiation pattern $H{( {\boldsymbol{k}} )_{max}}$. We remark that the far-field information of DPMMs (1−γ) is close to zero, which indicates that the generated radiation pattern is approximately omnidirectional, with the reflected energy almost uniformly spread in space. The low information property of DPMMs is preferred for stealth applications [8,27]. However, the fixed far-field information (1−γ) inevitably diminishes the flexibility of the radiation patterns. Consequently, radiation patterns with information values other than 1−γ cannot be realized by DPMMs, whereas these radiation patterns might be useful for information processing such as computational imaging. Additionally, it should be noted that the information invariance of DPMMs is rather intriguing, for which 1−γ might be the lower bound of radiation-pattern information when the size of metasurface is much greater than the wavelength squared of electromagnetic waves. Therefore, a further research to explore the information invariance of the chaotic radiation pattern is recommended.

## CONCLUSION

In summary, the presented theory establishes a quantitative framework to characterize the information processing capabilities of metasurfaces, which provides deeper physical insights into understanding metasurfaces from the information perspective, and offers new approaches to facilitate analysis and design of metasurfaces. The findings of this investigation are generally applicable in a wide range of spectra, and could help to lay the groundwork for future research into the regime of information metasurfaces. The proposed theory may also be applied in exploring information on cloaking systems [30] after making some deformations of the curved metasurface.

## METHODS

Under the normal incidence of the plane waves, the radiation function scattered by the metasurface can be expressed as [8]:

(22) \begin{eqnarray*}R(\theta ,\varphi ) &=& \Bigg| \sum\limits_{m = 1}^{{N_x}} {\sum\limits_{n = 1}^{{N_y}} {{c_{\mathit {ij}}}} }\! \exp \{ \mathit{jk}[a(m - 1)\nonumber\\ &&\times\sin\! \theta\! \cos\! \varphi + b(n {-} 1)\sin\! \theta\! \sin\! \varphi ]\} \Bigg|^2,\nonumber\\ \end{eqnarray*}

where ${c_{\mathit {ij}}} = {A_{\mathit {ij}}}{e^{\!\!j\!{\theta _{\mathit {ij}}}}}$ is the amplitude and phase response of the *ij*^th^ metasurface element. In addition, the coordinate transformation and normalization procedure ($R( {\theta ,\varphi } ) \to F\!( {{k_x},{k_y}}\! ) \to f\!( {{k_x},{k_y}}\! )$) should be adopted to obtain the normalized radiation pattern in *k*-space, where ${k_x} = k{\rm{sin\theta cos\varphi }}$, ${k_y} = k{\rm{sin\theta sin\varphi \ }}$and${\rm{\ }}f\!({{k_x}\!,{k_y}}\! ) = \ F\!( {{k_x}\!,{k_y}}\! )/\mathop \int\!\!\!\int \nolimits^ F( {{k_x}\!,{k_y}}\! )d{k_x}d{k_y}$. In this work, the intensity of the far-field radiation pattern is expressed as a discrete function ${g_{\!M}}( i\! )$, in which *M* stands for the number of discrete points in *k*-space. It should be noted that the radiation function is pre-normalized ($\mathop \sum \nolimits_{i = 1}^M {g_{\!M}}( i\! ) = 1$), such that the discrete version of the radiation-pattern information can be expressed as:

(23) \begin{eqnarray*}{I_2} &=& H{({\boldsymbol{k}})_{\max }} - H({\boldsymbol{k}}) = \mathop {\lim }\limits_{M \to \infty } \ln\! M\nonumber\\ && - \mathop {\lim }\limits_{M \to \infty } \sum\limits_{i = 1}^M { - {g_{\!M}}(i\!)} \cdot \ln [{g_{\!M}}(i\!)].\end{eqnarray*}

However, limited by the finite computational resources, the number of measurement points *M* cannot be chosen arbitrarily large. In this work, the term *M* is set as 1941, which is sufficiently large to characterize the information of the radiation patterns.

## Supplementary Material

nwz195_Supplemental_File
