# Supplementary material for: Information theory of metasurfaces
Source: Natl Sci Rev. 2019 Nov 27;7(3):561–71. doi: 10.1093/nsr/nwz195 (PMC8289013; doi:10.1093/nsr/nwz195)
Supplement: nwz195_Supplemental_File [file nwz195_supplemental_file.docx]

Supplementary Materials for

Information Theory of Metasurfaces

Haotian Wu^1^, Guo Dong Bai^1^, Shuo Liu^1^, Lianlin Li^2^, Xiang Wan^1^, Qiang Cheng^1^, and Tie Jun Cui^1*^

^1^State Key Laboratory of Millimeter Waves, Southeast University, Nanjing 210096, China

^2^ State Key Laboratory of Advanced Optical Communication Systems and Networks, Department of Electronics, Peking University, Beijing 100871, China

^1*^ Email: [tjcui@seu.edu.cn](mailto:tjcui@seu.edu.cn)

**This file includes:**

Detailed derivation of Equations

Supplementary Text Note

References 9 and 30

Figures. S1 to S2

**Detailed Derivations of Equations**

**1. Equation (4):**

where$c_{ij}={({A_{ij}^{2}}/{\sum_{i=1}^{N_{x}} \sum_{j=1}^{N_{y}} A_{ij}^{2}})}^{\frac{1}{2}}$

**2. Inequality (7):**

$$\begin{matrix} H(k)\text{=}H(f(k)) \\ \geq\ln(\pi\text{e})^{2}-H(\mathbf{r}) \\ \text{=ln}(\pi\text{e})^{2}-(H(r)_{\max}-I_{1}) \\ =\text{ln}(\pi\text{e})^{2}-\ln N_{x}N_{y}ab+\ln N_{x}N_{y}+\sum_{i=1}^{N_{x}} \sum_{j=1}^{N_{y}} (c_{ij}^{2}\times\ln c_{ij}^{2}) \\ =\ln\frac{\pi^{2}e^{2}}{ab}+\sum_{i=1}^{N_{x}} \sum_{j=1}^{N_{y}} (c_{ij}^{2}\times\ln c_{ij}^{2}) \end{matrix}$$

**3. Inequality (8):**

$$\begin{matrix} I_{2}=I(k)=H(k)_{\max}-H(k) \\ \leq\ln\pi k^{2}-[\ln\frac{\pi^{2}e^{2}}{ab}+\sum_{i=1}^{N_{x}} \sum_{j=1}^{N_{y}} (c_{ij}^{2}\times\ln c_{ij}^{2})] \\ =\ln\pi k^{2}-\ln\frac{\pi^{2}e^{2}}{ab}-\sum_{i=1}^{N_{x}} \sum_{j=1}^{N_{y}} (c_{ij}^{2}\times\ln c_{ij}^{2}) \\ =\ln\frac{abk^{2}}{\pi e^{2}}-\sum_{i=1}^{N_{x}} \sum_{j=1}^{N_{y}} c_{ij}^{2}\ln c_{ij}^{2} \end{matrix}$$

**4. Inequality (9)**

$$\begin{matrix} I_{1}\text{+}I_{2}\text{=}I(\mathbf{r})+I(k) \\ \leq\ln N_{x}N_{y}\text{+}\sum_{i=1}^{N_{x}} \sum_{j=1}^{N_{\text{y}}} c_{ij}^{2}\ln c_{ij}^{2}\text{+}\ln\frac{abk^{2}}{\pi e^{2}}-\sum_{i=1}^{N_{x}} \sum_{j=1}^{N_{y}} c_{ij}^{2}\ln c_{ij}^{2} \\ \text{=ln(}\frac{N_{x}N_{y}abk^{2}}{\pi e^{2}}\text{)} \\ \text{=ln(}\frac{4N_{x}N_{y}ab\pi^{2}}{\pi e^{2}\lambda^{2}}\text{)} \\ \text{=ln(}\frac{4\pi S}{e^{2}\lambda^{2}}\text{)} \end{matrix}$$

where $\lambda={2\pi}/k$, and $S=N_{x}{\times N}_{y}\times a\times b$.

**5. Inequality (15):**

$$\begin{matrix} S\geq\max(S^{i})\geq\max(\frac{e^{2}\lambda^{2}}{4\pi}e^{I_{1}^{i}+I_{2}^{i}})\geq\max(\frac{e^{2}\lambda^{2}}{4\pi}e^{I_{2}^{i}}) \\ \text{=}\frac{e^{2}\lambda^{2}}{4\pi}\max[\exp(\ln\pi k^{2}\text{+}\int\int f^{i}(k_{x},k_{y})\ln f^{i}(k_{x},k_{y})dk_{x}dk_{y})] \end{matrix}$$

where $S^{i}$ is the lower bound size of the $i^{th}$ radiation pattern, and $f^{i}$ is the $i^{th}$ radiation pattern.

**6. Inequality (21)**

$$\begin{matrix} I_{2}=H(\mathbf{p})_{\max}-H(\mathbf{p}) \\ =\lim_{M\to\infty} \ln M-\lim_{M\to\infty} \sum_{i=1}^{M} -g_{M}(i)\cdot\ln[g_{M}(i)] \\ =\lim_{M\to\infty} \ln M\text{+}\lim_{M\to\infty} \sum_{i=1}^{M} \text{(}-\frac{1}{M}\ln\frac{M-i+1}{M})\ln(-\frac{1}{M}\ln\frac{M-i+1}{M}) \\ =\lim_{M\to\infty} \ln M+\lim_{M\to\infty} \sum_{i=1}^{M} \text{(}-\frac{1}{M}\ln\frac{M-i+1}{M})\ln(\frac{1}{M})+\lim_{M\to\infty} \sum_{i=1}^{M} \text{(}-\frac{1}{M}\ln\frac{M-i+1}{M})\ln(-\ln\frac{M-i+1}{M}) \\ =\lim_{M\to\infty} \ln M+\ln(\frac{1}{M})+\lim_{M\to\infty} \sum_{i=1}^{M} \text{(}-\frac{1}{M}\ln\frac{M-i+1}{M})\ln(-\ln\frac{M-i+1}{M}) \\ =\lim_{M\to\infty} \sum_{i=1}^{M} \text{(}-\frac{1}{M}\ln\frac{M-i+1}{M})\ln(-\ln\frac{M-i+1}{M}) \\ =-\int_{0}^{1} \ln(1-x)\ln(-\ln(1-x))dx \\ =-\int_{0}^{1} \ln x\ln(-\ln x)dx \end{matrix}$$

Suppose that *x*=exp(-*t*), and *I*_2_ then can be derived as:

$$\begin{matrix} I_{2}\text{=}-\int_{0}^{1} \ln x\ln(-\ln x)dx \\ \text{=}\int_{0}^{\text{+}\infty} t\times\ln t\times\exp(-t)dt \end{matrix}$$

We now invoke the integration relation that (*28*):

$$\int_{0}^{\text{+}\infty} x^{v-1}e^{-ux}\ln xdx=\frac{1}{u^{v}}\Gamma(v)[\psi(v)-\ln u]$$

and substitute v=2 and u=1. Hence

$$\begin{matrix} I_{2}=\int_{0}^{\text{+}\infty} x^{v-1}e^{-ux}\ln xdx=\frac{1}{u^{v}}\Gamma(v)[\psi(v)-\ln u] \\ =\Gamma(2)[\psi(2)-\ln1] \\ =\Gamma(2)\psi(2)=(2-1)!\psi(2) \\ =\psi(2) \end{matrix}$$

where $\Gamma\left( t \right)$ is the gamma function, and $\psi(t)$ is defined as:

$$\psi(t)=\frac{d}{dt}\ln\Gamma(t)$$

which satisfies the relation that:

$$\psi(t)=\int_{0}^{1} \frac{x^{t-1}-1}{x-1}dx-\gamma$$

Then *I*_2_ can be further calculated as:

$$I_{2}=\psi(2)=1-\gamma$$

**Text Note: The Information (*I*_2_) and Image Entropy of Radiation Pattern**

The defined information of radiation pattern I_2_ of a metasurface is different from the previous established one. For instance, the previous definition of information entropy of the radiation pattern is based on the concept of ***image entropy***, as presented in Ref. (9), in which the image of the far-field pattern is divided into subsections, and intensities of adjacent subsections are labeled by two numbers (i, j) from (0, 0) to (255, 255). Accordingly, the image entropy of the radiation pattern can be calculated as: $H^{image}=-\sum p_{ij}lnp_{ij}$, where $p_{ij}$ is the probability of the intensity distribution (i, j) of adjacent subsections. To quantitatively compare the defined information I_2_ and image entropy (*9*) of the radiation pattern, we consider a set of disordered phase modulated metasurfaces, which is generated by randomly flip the phase of metasurface element from 0 to π (or π to 0) with multiple iterations, as shown in Fig. S2. The metasurface sample is composed of 30×30 elements, in which the size of the element is set as 1/6λ×1/6λ. The numerical results of the proposed information I_2_ and the image entropy (*9*) with respect to different radiation patterns are presented in Fig. S2c.

**REFERENCES**

9. Cui TJ, Liu S, Li LL, Information entropy of coding metasurface. *Light-Sci. Appl* 2016; **5**: e16172.

30. I. S. Granshteyn, I. M. Ryzhik. Table of Integrals, Series, and Products. *Academic Press* 2007; 7^th^ edition.


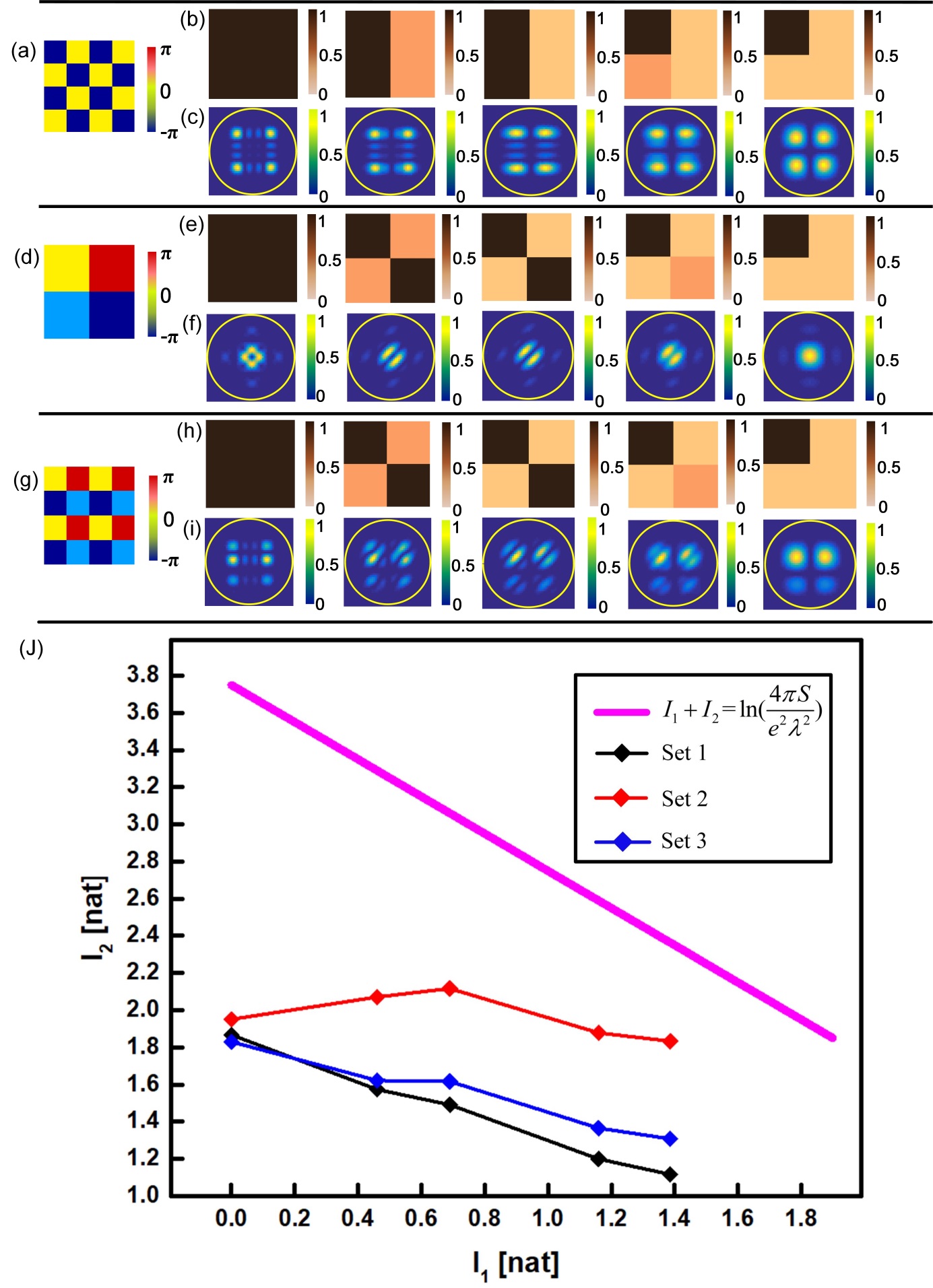


**Figure S1.** Phase (a, d, g) and amplitude (b, e, h) distributions of three sets of metasurface samples. (c, f, i) Normalized radiation patterns generated by the three sets of metasurface samples. (j) Calculated results of the information relation between the metasurface samples and their radiation patterns, and the upper bound.


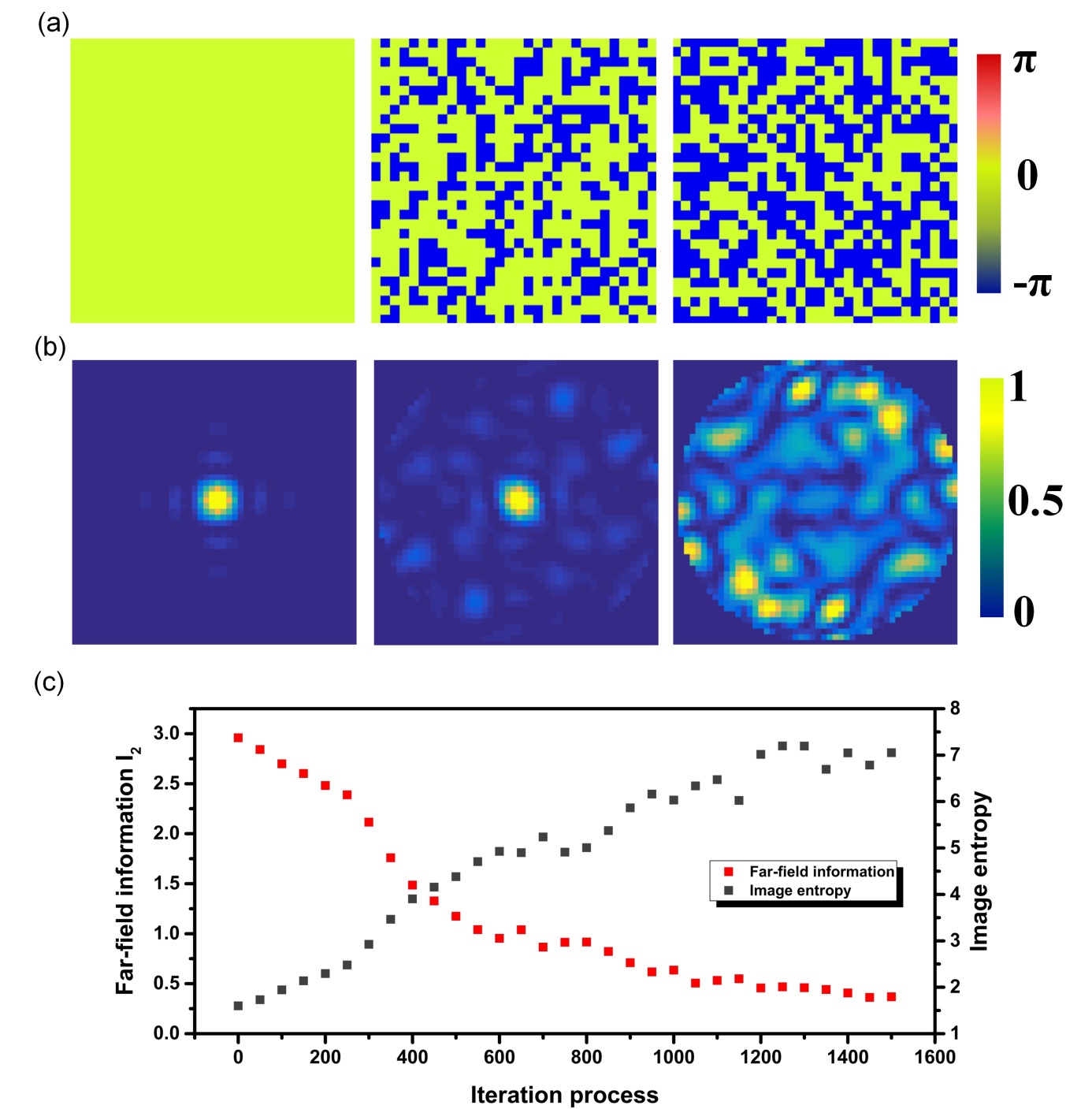


**Figure** **S2.** (a, b) Disordered-phase modulated metasurfaces at different diffusion states and the corresponding far-field radiation patterns. (c) The calculated far-field information *I*_2_ and image entropy of the radiation patterns.
